# Liver injury in critically ill patients with COVID-19: a case series

**DOI:** 10.1186/s13054-020-02924-4

**Published:** 2020-05-05

**Authors:** Filipe S. Cardoso, Rui Pereira, Nuno Germano

**Affiliations:** Intensive Care Unit, Curry Cabral Hospital, R. Beneficiencia N8, 1050-099 Lisbon, Portugal

Dear Editor,

Almost all reports on liver injury in patients with 2019 coronavirus disease (COVID-19) found blood liver tests to be frequently abnormal, especially in patients with more severe disease, but with substantial heterogeneity [1]. Moreover, blood liver tests’ abnormalities were frequently thought to be of doubtful clinical value.

Most studies have described blood liver tests in a single time point, usually at inclusion [2, 3]. Therefore, we used our case series of the first 20 consecutive patients with COVID-19 admitted to the intensive care unit (ICU) at Curry Cabral Hospital in Lisbon, Portugal, from March 10, 2020, onwards, to describe the temporal evolution of blood liver tests.

Median (interquartile range (IQR)) age was 67 (52–74) years with 18 (90%) males (Table 1). Median (IQR) time from symptom onset to hospital admission was 7.5 (5.5–8.5) days, and median time from hospital admission to ICU admission was 1.1 (0.7–2.1) days. All patients required invasive mechanical ventilation on ICU admission. Median (IQR) sequential organ failure assessment (SOFA) score on ICU admission and peak was 8 (7–9) and 9 (8–11), respectively. As of April 10, following a median (IQR) of 21.5 (11.2–25.4) days post ICU admission, 3 (15%) patients died of multi-organ failure, 14 (70%) were discharged to the ward, and 3 (15%) remained in the ICU.

**Table 1:** Baseline characteristics and outcomes of patients

Characteristic	Median (IQR) or *n* (%)
Age (years)	67 (52–74)
Sex (male)	18 (90%)
BMI (kg/m^2^)	29 (26–32)
Parameters on ICU admission	
P_a_O_2_/FiO_2_	138 (128–163)
Lactate (mmol/L)	1.1 (0.8–1.2)
Creatinine (mg/dL)	1.11 (1.04–1.26)
Organ support during ICU stay	
Invasive mechanical ventilation	20 (100%)
Vasopressors	19 (95%)
Renal replacement therapy	7 (35%)
SOFA score	
ICU admission	8 (7–9)
Peak	9 (8–11)
ICU discharge	3 (2–6)
APACHEII score	18 (14–21)
ICU mortality	3 (15%)
Hospital mortality	3 (15%)
ICU length of stay (days)	10.3 (8.0–12.3)
Hospital length of stay (days)	22.4 (14.1–26.7)

*IQR* interquartile range, *BMI* body mass index, *P*_*a*_*O*_*2*_*/FiO2* oxygen partial pressure/oxygen inspired fraction, *SOFA* Sequential Organ Failure Assessment, *ICU* intensive care unit, *APACHEII* Acute Physiology and Chronic Health Evaluation II

No patient had documented liver disease prior to hospitalization. During the first 10 days post ICU admission, all patients had at least one abnormal blood liver test. Overtime, only median gamma-glutamiltranspeptidase (GGT), alanine transferase (ALT), and aspartate transferase (AST) showed any increase from upper limit of normal (ULN) and only median GGT had a ≥ 3 fold increase from ULN (Table 2). Median peak GGT was on day 8 post ICU admission. Patients with peak C-reactive protein ≥ 250 mg/L (day 4 post ICU admission) had higher but non-significant median peak GGT (298 vs. 125 IU/L), ALT (101 vs. 42 IU/L), or AST (72 vs. 57 IU/L) than others (*P* > 0.50 for all comparisons).

**Table 2:** Temporal evolution of median levels of blood tests

Test (upper limit of normal)	H adm	ICU adm	D2	D3	D4	D5	D6	D7	D8	D9	D10
INR (≤ 1.2)	1.18	1.17	1.20	1.16	1.17	1.18	1.17	1.15	1.17	1.17	1.16
Bilirubin (≤ 1.2 mg/dL)	0.65	0.80	1.01	1.16	0.80	0.86	1.05	0.92	1.15	0.87	0.97
ALP (≤ 150 IU/L)	61	59	61	74	83	89	101	95	116	125	111
GGT (≤ 64 IU/L)	55	52	53	66	92	73	102	214	237	211	225
ALT (≤ 55 IU/L)	31	30	33	31	43	48	56	67	82	55	72
AST (≤ 34 IU/L)	51	51	51	44	49	57	69	62	60	53	46
CRP (≤ 5 mg/L)	176	207	239	257	271	258	198	153	97	76	74

*H adm* hospital admission, *ICU adm* intensive care unit admission, *INR* international normalized ratio, *ALP* alkaline phosphatase, *GGT* gamma-glutamiltranspeptidase, *ALT* alanine aminotransferase, *AST* aspartate aminotransferase, *CRP* C-reactive protein

In our case series, liver injury was frequent but generally transient and non-severe. While synthetic function was largely preserved, late cholestasis was frequently observed. Cholestasis may have been associated to the critical illness itself (inflammation), parenteral nutrition (only 2 patients required parenteral nutrition), or drug toxicity (all patients were on antibiotics, for example ceftriaxone, amoxicillin-clavulanate, or azithromycin) [4, 5]. While cholestasis could have been multifactorial, the differential diagnosis was not easy to perform with precision. Overall, attention to modifiable factors, such as control of inflammation, timing of parenteral nutrition, and avoiding drugs with worse liver toxicity profile, may be important to prevent cholestasis progression in these patients. Further studies are needed to understand liver injury in critically ill patients with COVID-19, especially if there is any direct viral effect on the liver cells.

## Data Availability

The datasets generated and/or analyzed during the current study are not publicly available due to confidentiality but are available from the corresponding author on reasonable request.

## Abbreviations

ALT: Alanine transferase
ALP: Alkaline phosphatase
AST: Aspartate transferase
COVID-19: 2019 coronavirus disease
CRP: C-reactive protein
ICU: Intensive care unit
IMV: Invasive mechanical ventilation
INR: International normalized ratio
IQR: Interquartile range
GGT: Gamma-glutamiltranspeptidase
